# Supplementary material for: CD47 and IFT57 Are Colinear Genes That Are Highly Coexpressed in Most Cancers and Exhibit Parallel Cancer-Specific Correlations with Survival
Source: Int J Mol Sci. 2024 Aug 17;25(16):8956. doi: 10.3390/ijms25168956 (PMC11354933; doi:10.3390/ijms25168956)
Supplement: Supplementary file 1 [file ijms-25-08956-s001.zip › DataS2.pdf]

INGENUITY
PATHWAY ANALYSIS

Analysis Name: Volcano_Summary_IFT57KO_New - 2024-08-05 01:57 PM
Analysis Creation Date: 2024-08-05
Build version: exported
Content version: 111725566 (Release Date: 2024-03-21)

Experiment Metadata

Name	Value

Analysis Settings

Top Canonical Pathways

Name	p-value	Overlap
**Axonal Guidance Signaling**	5.51E-09	10.0 % 51/510
**Integrin cell surface interactions**	8.92E-09	21.2 % 18/85
**Extracellular matrix organization**	1.53E-06	16.0 % 17/106
**Degradation of the extracellular matrix**	1.41E-05	18.2 % 12/66
**Collagen biosynthesis and modifying enzymes**	1.66E-05	17.9 % 12/67

(c) 2000-2024 QIAGEN. All rights reserved.

Top Upstream Regulators

Upstream Regulators

Name	p-value	Predicted Activation
**TNF**	8.91E-22	Inhibited
**TGFB1**	2.46E-17	Inhibited
**IL1B**	4.18E-17	Inhibited
**PGR**	7.39E-17	
**beta-estradiol**	1.91E-16	Inhibited

Causal Network

Name	p-value	Predicted Activation
**dantrolene**	3.67E-23	Activated
**IL13-downregulated genes for extracellular proteins**	6.83E-23	Inhibited
**lenalidomide**	3.80E-22	
**malondialdehyde**	3.89E-22	Inhibited
**levonorgestrel**	5.84E-22	Activated

Top Diseases and Bio Functions

(c) 2000-2024 QIAGEN. All rights reserved.

INGENUITY PATHWAY ANALYSIS

Diseases and Disorders

Name	p-value range	# Molecules
**Cancer**	1.33E-03 - 2.56E-62	980
**Organismal Injury and Abnormalities**	1.33E-03 - 2.56E-62	996
**Gastrointestinal Disease**	1.30E-03 - 7.64E-46	907
**Endocrine System Disorders**	1.10E-03 - 1.40E-35	872
**Reproductive System Disease**	1.20E-03 - 3.43E-35	765

Molecular and Cellular Functions

Name	p-value range	# Molecules
**Cellular Movement**	1.31E-03 - 2.34E-16	334
**Cellular Assembly and Organization**	1.09E-03 - 2.31E-09	258
**Cellular Function and Maintenance**	1.09E-03 - 2.31E-09	216
**Cellular Development**	1.33E-03 - 3.55E-09	383
**Cellular Growth and Proliferation**	1.33E-03 - 3.55E-09	372

Physiological System Development and Function

Name	p-value range	# Molecules
**Cardiovascular System Development and Function**	1.30E-03 - 1.43E-13	208
**Organismal Development**	1.30E-03 - 1.43E-13	353

(c) 2000-2024 QIAGEN. All rights reserved.

INGENUITY PATHWAY ANALYSIS

**Embryonic Development**	1.30E-03 - 4.27E-10	247
**Tissue Development**	1.32E-03 - 9.68E-10	343
**Hematological System Development and Function**	1.24E-03 - 6.08E-09	215

Top Tox Functions

Assays: Clinical Chemistry and Hematology

Name	p-value range	# Molecules
**Increased Levels of ALT**	1.00E00 - 3.34E-03	5
**Increased Levels of Alkaline Phosphatase**	1.83E-02 - 1.83E-02	8
**Increased Levels of Hematocrit**	2.78E-02 - 2.78E-02	9
**Increased Levels of Bilirubin**	8.07E-02 - 8.07E-02	1
**Increased Levels of Albumin**	3.96E-01 - 2.55E-01	2

Cardiotoxicity

Name	p-value range	# Molecules
**Cardiac Dysfunction**	1.00E00 - 8.45E-07	55
**Cardiac Infarction**	2.16E-01 - 5.14E-06	36
**Cardiac Arteriopathy**	5.51E-01 - 1.92E-05	38
**Congenital Heart Anomaly**	1.00E00 - 2.37E-05	39
**Cardiac Enlargement**	1.00E00 - 4.20E-05	67

(c) 2000-2024 QIAGEN. All rights reserved.

INGENUITY PATHWAY ANALYSIS

Hepatotoxicity

Name	p-value range	# Molecules
**Liver Hyperplasia/Hyperproliferation**	1.00E00 - 1.14E-23	516
**Liver Fibrosis**	1.00E00 - 4.28E-05	46
**Hepatocellular carcinoma**	1.00E00 - 7.82E-05	153
**Liver Cirrhosis**	3.96E-01 - 1.04E-04	32
**Liver Inflammation/Hepatitis**	1.00E00 - 2.11E-04	41

Nephrotoxicity

Name	p-value range	# Molecules
**Kidney Failure**	5.31E-01 - 6.13E-04	25
**Renal Damage**	1.00E00 - 1.19E-03	33
**Renal Inflammation**	6.20E-01 - 1.40E-03	38
**Renal Nephritis**	6.20E-01 - 1.40E-03	38
**Renal Tubule Injury**	1.00E00 - 1.53E-03	15

Top Regulator Effect Networks

ID	Regulators	Disease & Functions	Consistency Score
**1**	CREBBP,HYAL1,INS,INSIG1,RORA,TRIM24,TWIST2	Abnormality of left ventricle,Dysfunction of heart (+3 more)	7.924
**2**	INSIG1,JAK (family),RC3H1,TRIM24	Abnormality of left ventricle (+7 more)	5.8
**3**	IFNB1,MAPK1,VTN	Remodeling of blood vessel,Systolic pressure	4.243

(c) 2000-2024 QIAGEN. All rights reserved.

INGENUITY PATHWAY ANALYSIS

4	CGAS,HYAL1,IL17F,RASSF1,RYK	Abnormal aortic valve physiology,Cardiomyopathy (+2 more)	4.2
5	BANF1,CHROMR,FGF21,IFNL1,INTERFERON ALPHA (family) (+8 more)	Differentiation of neuronal progenitor cells (+1 more)	4.082

Top Networks

ID	Associated Network Functions	Score
1	Cellular Assembly and Organization, Developmental Disorder, Organismal Injury and Abnormalities	45
2	Carbohydrate Metabolism, Post-Translational Modification, Developmental Disorder	36
3	Developmental Disorder, Hereditary Disorder, Neurological Disease	36
4	Cell Death and Survival, Organismal Injury and Abnormalities, Cellular Assembly and Organization	36
5	Endocrine System Disorders, Gastrointestinal Disease, Metabolic Disease	34

(c) 2000-2024 QIAGEN. All rights reserved.

Top Tox Lists

Name	p-value	Overlap
**Hepatic Fibrosis**	2.67E-05	9.1 % 32/352
**VDR/RXR Activation**	7.92E-05	15.4 % 12/78
**Persistent Renal Ischemia-Reperfusion Injury (Mouse)**	1.22E-03	20.0 % 6/30
**PXR/RXR Activation**	1.48E-03	13.6 % 9/66
**Irreversible Glomerulonephritis Biomarker Panel (Rat)**	1.52E-03	30.8 % 4/13

Top My Lists

Top My Pathways

Top ML Disease Pathways

Name	p-value	Overlap
**Aneurysm**	2.85E-05	22.5 % 9/40
**Infarction of cerebrum**	1.92E-04	20.0 % 8/40
**Right ventricular abnormality**	3.24E-04	18.6 % 8/43
**Ventricular septal defect**	4.48E-04	17.8 % 8/45
**Heart septal defect**	4.48E-04	17.8 % 8/45

(c) 2000-2024 QIAGEN. All rights reserved.

INGENUITY PATHWAY ANALYSIS

Top Analysis-Ready Molecules

Expr Fold Change

Molecules	Expr. Value	Chart
AUTS2	↑ 183.243	
ENOX1	↑ 75.826	
HOOK1	↑ 63.864	
EBF2	↑ 60.560	
LINC00652	↑ 44.607	
ERG	↑ 44.009	
LINC00623	↑ 40.518	
SLC1A3	↑ 39.927	
KCTD8	↑ 35.194	
TNFRSF19	↑ 27.858	

Expr Fold Change

Molecules	Expr. Value	Chart
APBB1IP	↓ -908.510	
TMEM132D	↓ -405.499	
BEX1	↓ -201.322	
PTPRD	↓ -161.523	
COL14A1	↓ -115.697	

(c) 2000-2024 QIAGEN. All rights reserved.

INGENUITY PATHWAY ANALYSIS

**ECHDC3**	↓ -109.502
**SCIN**	↓ -94.067
**PCDHGB5**	↓ -83.891
**KRT19**	↓ -78.301
**SERPINB9**	↓ -71.438

(c) 2000-2024 QIAGEN. All rights reserved.

INGENUITY PATHWAY ANALYSIS
