# Supplementary material for: CD47 and IFT57 Are Colinear Genes That Are Highly Coexpressed in Most Cancers and Exhibit Parallel Cancer-Specific Correlations with Survival
Source: Int J Mol Sci. 2024 Aug 17;25(16):8956. doi: 10.3390/ijms25168956 (PMC11354933; doi:10.3390/ijms25168956)
Supplement: Supplementary file 1 [file ijms-25-08956-s001.zip › Kun IFT57 cancers- 8-7-2024 supplement.pdf]

Data Supplement for:

***CD47* and *IFT57* Are Colinear Genes That Are Highly Coexpressed in Most Cancers and Exhibit Parallel Cancer-Specific Correlations with Survival**

Kun Dong, Raghib Nihal, Thomas J. Meyer, Satya P. Singh, Sukhbir Kaur and David D. Roberts

Supplemental Tables

Table S1. Validation in TCGA papillary thyroid carcinoma tumors of IFT57- and CD47-coregulated genes identified by RNAseq in 8505C anaplastic thyroid carcinoma cells.

Gene	Log_2FC IFT57/WT in 8505C	IFT57 coexpression (Spearman)	p-value	CD47 coexpression (Spearman)	p-value
PER3	3.61	0.136	2.04×10^{-3}	0.184	3.06×10^{-5}
HSPB8	-14.27	-0.353	2.04×10^{-16}	-0.483	4.57×10^{-31}
VCX3A	-35.23	-0.102	0.0213	-0.281	1.01×10^{-10}
ANK3	3.08	0.104	0.0193	-0.142	1.34×10^{-3}
HES7	-2.11	0.233	1.11×10^{-7}	0.121	6.11×10^{-3}
BMP8B	-3.50	-0.112	0.0117	-0.311	6.48×10^{-13}
ZDHHC23	2.31	-0.212	1.44×10^{-6}	-0.312	6.38×10^{-13}
RAB6B	3.44	-0.264	1.46×10^{-9}	-0.466	7.78×10^{-29}
AK4	2.34	-0.0953	0.0315	0.272	4.27×10^{-10}
CADM1	-6.06	0.517	3.99×10^{-36}	0.578	9.35×10^{-47}
EEF1A2	-3.16	-0.193	1.20×10^{-5}	-0.163	2.29×10^{-4}

Table S2. Validation of 46 CD47-specific genes identified by RNAseq in 8505C anaplastic thyroid carcinoma cells for selective coexpression with CD47 in TCGA papillary thyroid carcinoma tumors.

	8505C CD47 knockdown		Coexpression in TCGA papillary thyroid carcinomas			
Gene	CD47Log2FC	pvalue	IFT57 coexpression	p-value	CD47 coexpression	p-value
AKR1C3	-1.61637	0.01568		ns	-0.361	4.13E-17
AMIGO2	-0.66245	1.18E-05	0.269	6.77E-10	0.542	2.81E-40
APOE	-0.85111	0.001714	0.332	1.38E-14	-0.206	2.67E-06
ARFGEF3	0.761371	2.16E-04	-0.241	3.74E-08	-0.111	0.012
BCL2A1	-0.74216	2.83E-04		ns	0.298	7.28E-12
CA9	0.582376	0.001779		ns	-0.206	2.67E-06
CCDC159	-0.92743	0.003092		ns	-0.175	7.25E-05
CCSER1	0.928349	5.40E-04	-0.313	4.97E-13	-0.399	6.77E-21
CHMP4C	0.596842	0.0069		ns	0.27	6.35E-10
CORO2B	-0.82321	8.63E-06		ns	-0.38	5.71E-19
CRABP2	-0.62163	0.011622	0.119	7.10E-03	0.31	8.02E-13
FBXO30-DT	-1.55797	0.021771		ns	-0.193	1.18E-05
FST	-0.75089	4.26E-04		ns	0.169	1.22E-04
GAD1	-0.59571	0.011548	-0.258	3.54E-09	-0.173	8.96E-05
GCNA	-0.71252	0.00157		ns	0.154	5.01E-04
GGT5	-0.62966	0.004045	0.221	4.48E-07	0.246	1.76E-08
GTSF1	-1.18309	2.32E-04		ns	0.201	4.91E-06
HCG4	0.687265	0.005206		ns	-0.249	1.17E-08
HSPA6	-1.00129	0.026259	-0.128	3.86E-03	0.0986	0.0261
KLHL4	0.808081	0.0015		ns	0.127	4.14E-03
KYNU	-0.75371	2.18E-04		ns	0.21	1.69E-06
LHFPL5	0.826917	0.006584		ns	0.156	4.15E-04
LRP3	1.221539	2.33E-04	-0.125	4.61E-03	-0.22	5.31E-07
MBNL3	0.652574	0.006189		ns	0.0906	0.041
MLPH	-0.5921	2.51E-04	-0.312	6.32E-13	-0.0891	0.0445
NCAM2	0.718508	1.40E-04	-0.0986	0.0262	0.184	2.88E-05
PIK3R6	0.729238	0.010035		ns	0.243	2.76E-08
PLAAT4	-0.83704	0.003349	0.0942	0.0337	0.26	2.54E-09
PPFIA4	1.409059	0.002462	0.183	3.16E-05	0.242	3.11E-08
PTPN6	-0.6604	0.0014		ns	0.119	6.98E-03
RGPD6	1.150201	0.021705	0.123	5.40E-03	0.203	3.79E-06
RPLP0P2	-0.75753	0.001942		ns	0.169	1.23E-04
SECTM1	-1.01307	5.57E-04		ns	0.169	1.24E-04
SLC15A3	-1.07289	0.001684		ns	0.441	1.37E-25
SLC6A15	0.866978	0.007135		ns	-0.176	6.67E-05
SNX10	0.640495	2.35E-04		ns	0.174	8.28E-05
SRPX	-0.73029	6.04E-07		ns	0.101	0.0231
SULF1	0.760421	3.82E-07	0.114	9.90E-03	0.275	2.74E-10
TATDN2P2	0.654693	0.039005		ns	-0.287	3.94E-11
TEN1-CDK3	-0.65401	0.01132	-0.0912	0.0397	-0.167	1.55E-04
TMEM215	-1.02887	2.68E-04	0.284	7.24E-11	0.223	3.78E-07
TNFSF15	-0.76755	0.007563		ns	0.259	3.13E-09
TNIK	-0.86671	3.99E-05		ns	0.226	2.57E-07
WNT5A	-0.7173	0.003739	0.239	4.68E-08	0.509	5.93E-35
ZC3H6	0.629249	5.80E-04	0.191	1.46E-05	0.174	8.22E-05
ZNF471	0.634998	0.00536		ns	0.113	0.0108

Supplemental Figure Legends

Figure S1. Conserved colinear microsynteny of *CD47* with *IFT57* and correlation of CD47 mRNA expression with cancer survival. (**a**) Colinearity of *IFT57* and *CD47* genes in representative mammalian, avian, and reptilian genomes. (**b**) Comparison of IFT57 mRNA expression across tumor types in TCGA. (**c**) Overall survival for TCGA lung squamous adenocarcinomas segregated based on high versus low CD47 mRNA expression partitioned using an optimized expression cutoff. (**d**) Overall survival for TCGA low grade gliomas based on CD47 mRNA expression partitioned using an optimized expression cutoff.

Figure S2. Association of IFT57and CD47 mRNA expression with cancer survival. (**a,b**) Overall survival for lung squamous carcinomas with high versus low CD47 or IFT57 mRNA expression using a median cutoff (n = 482). (**c,d**) Overall survival for low grade gliomas with high versus low CD47 or IFT57 mRNA expression using a median cutoff (n = 514). (**e,f**) Overall survival for lung adenocarcinomas with high versus low CD47 or IFT57 mRNA expression using a median cutoff (n = 478).

Figure S3. Association of IFT57and CD47 mRNA expression with cancer survival. (**a**) Overall survival for TCGA head and neck squamous carcinoma tumors segregated based on CD47 or IFT57 mRNA expression greater than or less than the mean (n = 522). (**b**) Overall survival for TCGA breast invasive carcinoma tumors segregated based on CD47 or IFT57 mRNA expression greater than or less than the mean (n = 1100). (**c**) Overall survival for TCGA uveal melanoma tumors segregated based on CD47 or IFT57 mRNA expression greater than or less than the mean (n = 80).

Figure S4. CD47 and IFT57 are coexpressed in thyroid carcinoma cells, but IFT57 does not regulate CD47 expression. (**a**) Coexpression of CD47 and IFT57 in thyroid carcinoma cell lines in CCLE. (**b**) Effect of IFT57 siRNA knockdown on IFT57 and CD47 mRNA expression in 8505C cells. (**c**) Effect of IFT57 siRNA knockdown on IFT57 protein expression in 8505C cells. (**d**) Effect of transient over-expression of IFT57 in 8505C cells on short and long CD47 and IFT57 mRNA expression (** = $p \leq 0.01$, *** = $p \leq 0.001$).

Figure S5. Validation of bulk RNA sequencing analysis of WT and IFT57 mutants of the 8505C anaplastic thyroid carcinoma cell line. (**a**) Pathway analysis of IFT57-dependent genes in two independent knockdown clones was performed using ShinyGO 0.77, South Dakota State University. (**b,c**) Confirmation of IFT57-dependent fibronectin (FN1) expression. (**b**) Expression of FN1 mRNA in WT 8505C and the sg2-B6 and sg1F4 IFT57 knockdown mutants (mean ± SD, n = 3) (** = $p \leq 0.01$, *** = $p \leq 0.001$). (**c**) Expression of fibronectin (FN1) protein in WT 8505C and the sg2-B6 and sg1F4 IFT57 knockdown mutants. (**d**) Venn diagram showing overlap between IFT57-dependent genes identified in thyroid carcinoma cells and CD47-dependent genes previously identified in mouse CD8 T cells (GSE239430). (**e**) Effect of the CD47 ligand thrombospondin-1 on CRACD mRNA expression in parental 8505C cells (WT), the CD47 knockdown pool, and the indicated IFT57 knockdown clones. (**f**) Effect of the CD47 function blocking antibody B6H12 on CRACD mRNA expression in 8505C cells.

Figure S6. Correlations of IFT57-dependent mRNAs with survival in TCGA papillary thyroid

carcinomas. (**a**) Overall survival for tumors segregated based on RNF180 mRNA expression greater than or less than the mean (n = 509). (**b**) Disease free survival for tumors segregated based on RNF180 mRNA expression greater than or less than the mean (n = 509). (c) Overall survival for tumors segregated based on NMB mRNA expression greater than or less than the mean (n = 509). (d) Disease free survival for tumors segregated based on NMB mRNA expression greater than or less than the mean (n = 509).

Figure S7. Guide RNAs and PCR primers. (**a**) guide RNA sequences used for CRISPR targeting of IFT57 and CD47. (b). Primers for quantitative RT-PCR.

Figure S1

a

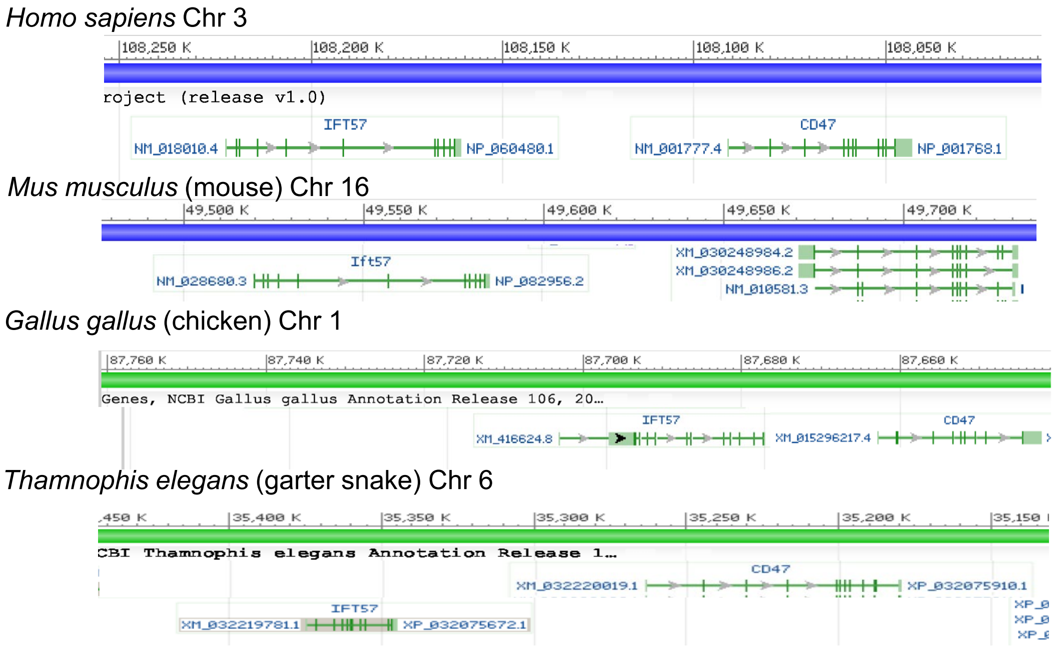

b

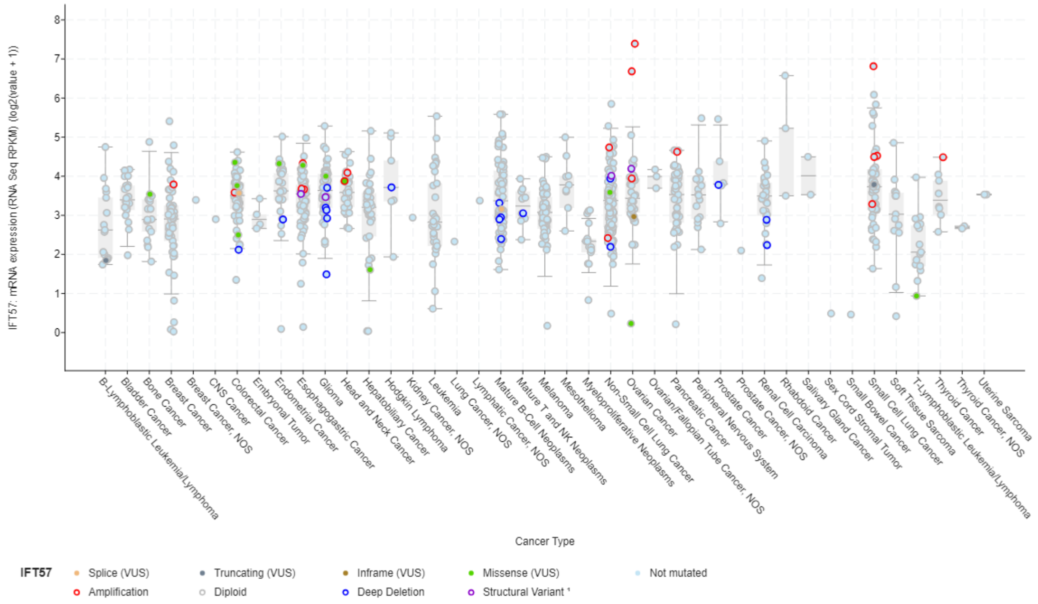

c

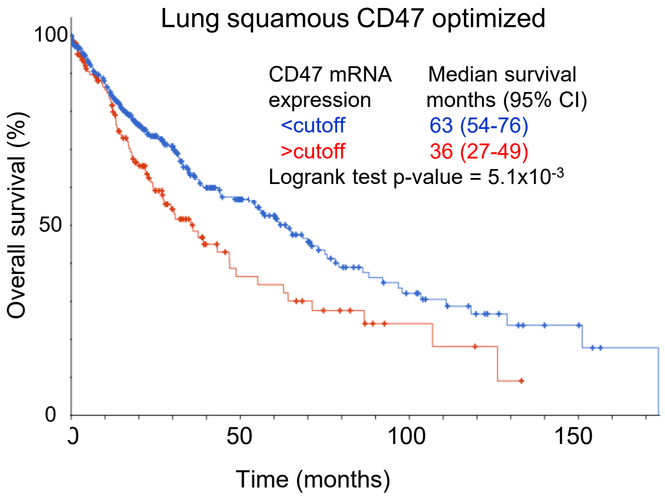

d

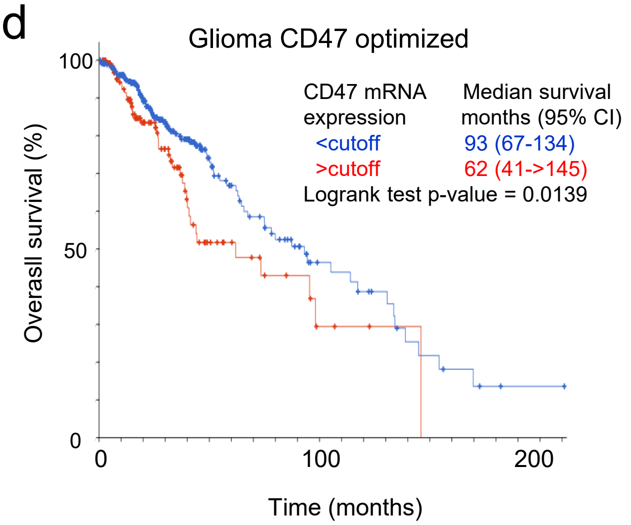

Figure S2

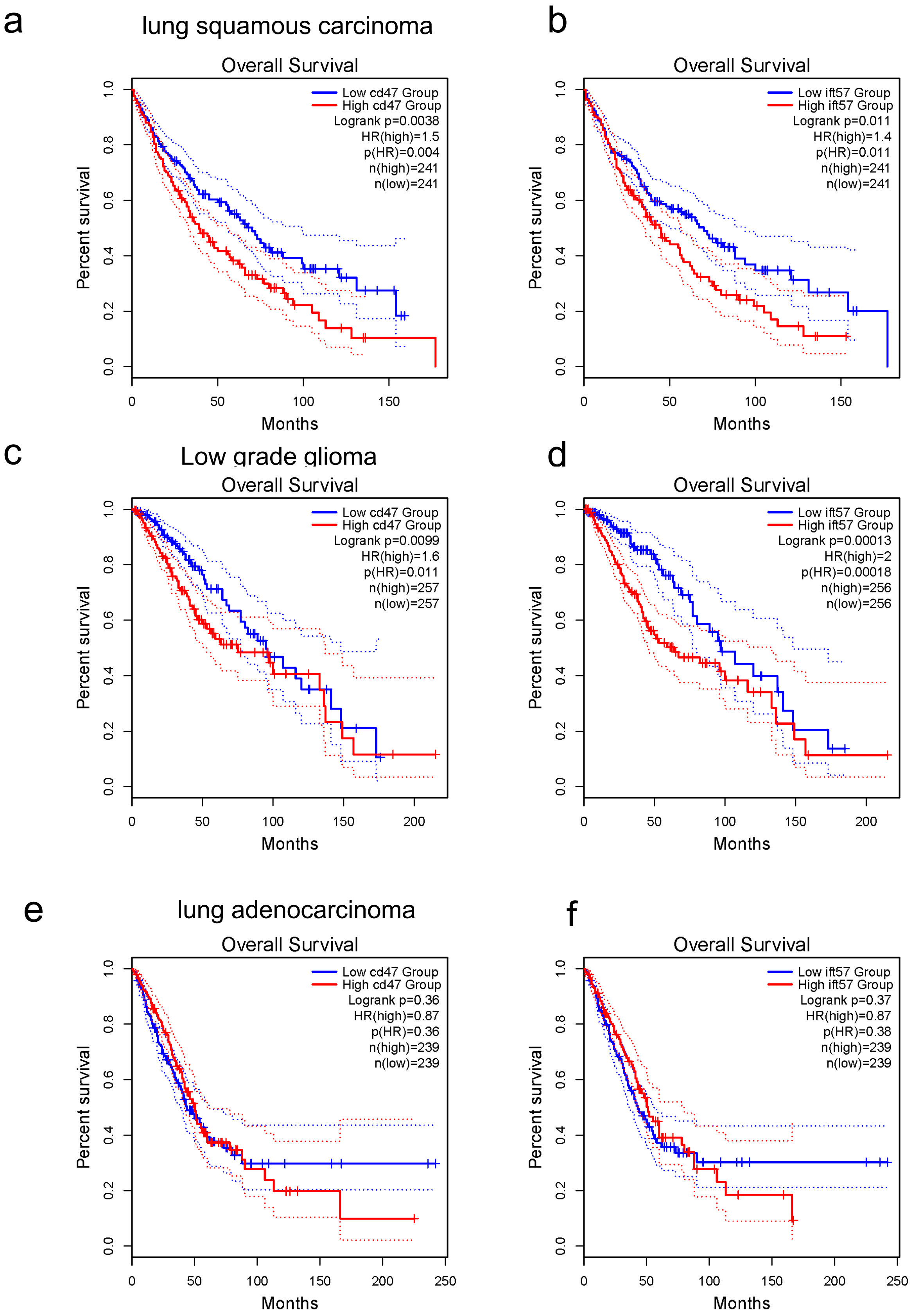
a
lung squamous carcinoma
Overall Survival
Low cd47 Group
High cd47 Group
Logrank p=0.0038
HR(high)=1.5
p(HR)=0.004
n(high)=241
n(low)=241
Percent survival
Months
b
Overall Survival
Low ift57 Group
High ift57 Group
Logrank p=0.011
HR(high)=1.4
p(HR)=0.011
n(high)=241
n(low)=241
Percent survival
Months
c
Low grade glioma
Overall Survival
Low cd47 Group
High cd47 Group
Logrank p=0.0099
HR(high)=1.6
p(HR)=0.011
n(high)=257
n(low)=257
Percent survival
Months
d
Overall Survival
Low ift57 Group
High ift57 Group
Logrank p=0.00013
HR(high)=2
p(HR)=0.00018
n(high)=256
n(low)=256
Percent survival
Months
e
lung adenocarcinoma
Overall Survival
Low cd47 Group
High cd47 Group
Logrank p=0.36
HR(high)=0.87
p(HR)=0.36
n(high)=239
n(low)=239
Percent survival
Months
f
Overall Survival
Low ift57 Group
High ift57 Group
Logrank p=0.37
HR(high)=0.87
p(HR)=0.38
n(high)=239
n(low)=239
Percent survival
Months

Figure S3

a Head and neck squamous carcinoma

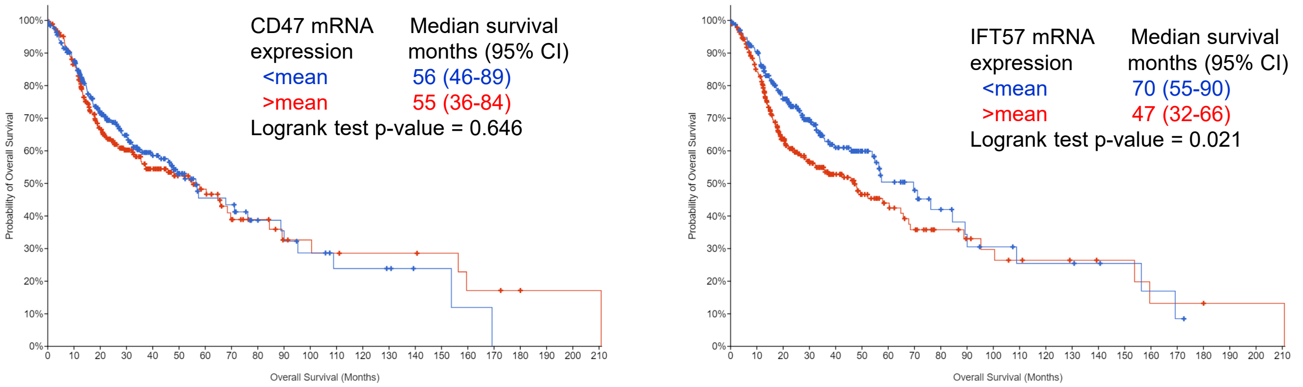

b Breast carcinoma

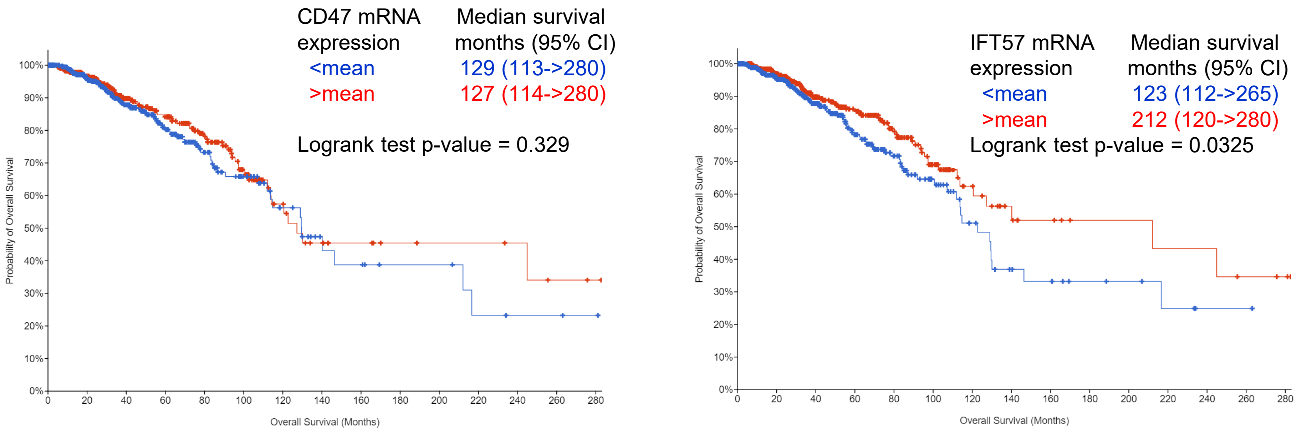

c Uveal melanoma

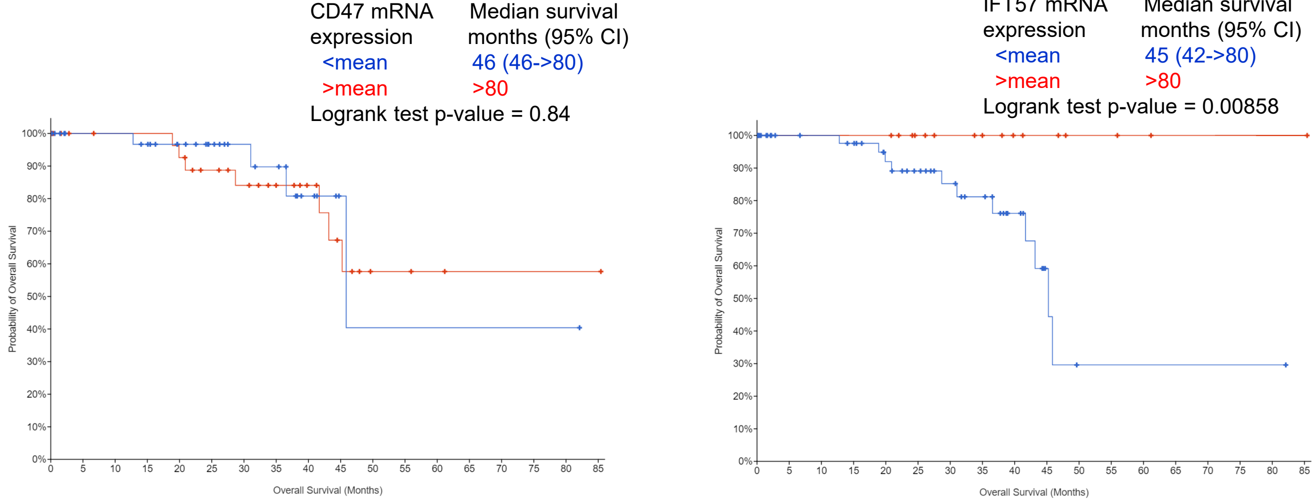

Figure S4

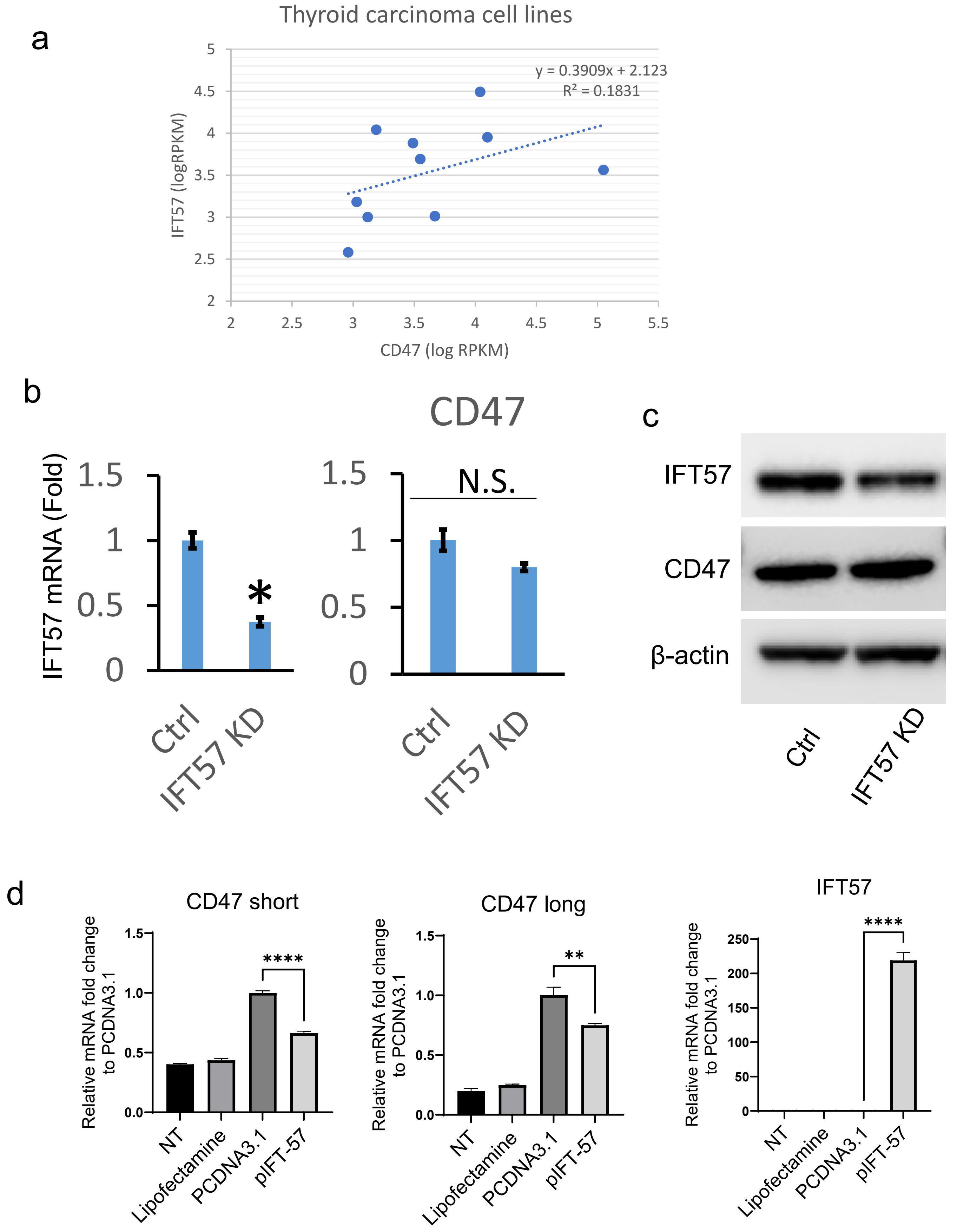

a
Thyroid carcinoma cell lines
y = 0.3909x + 2.123
R² = 0.1831
IFT57 (logRPKM)
CD47 (log RPKM)
b
CD47
IFT57 mRNA (Fold)
N.S.
*
Ctrl
IFT57 KD
c
IFT57
CD47
β-actin
Ctrl
IFT57 KD
d
CD47 short
CD47 long
IFT57
Relative mRNA fold change to PCDNA3.1
****
**
****
NT
Lipofectamine
PCDNA3.1
pIFT-57

Figure S5

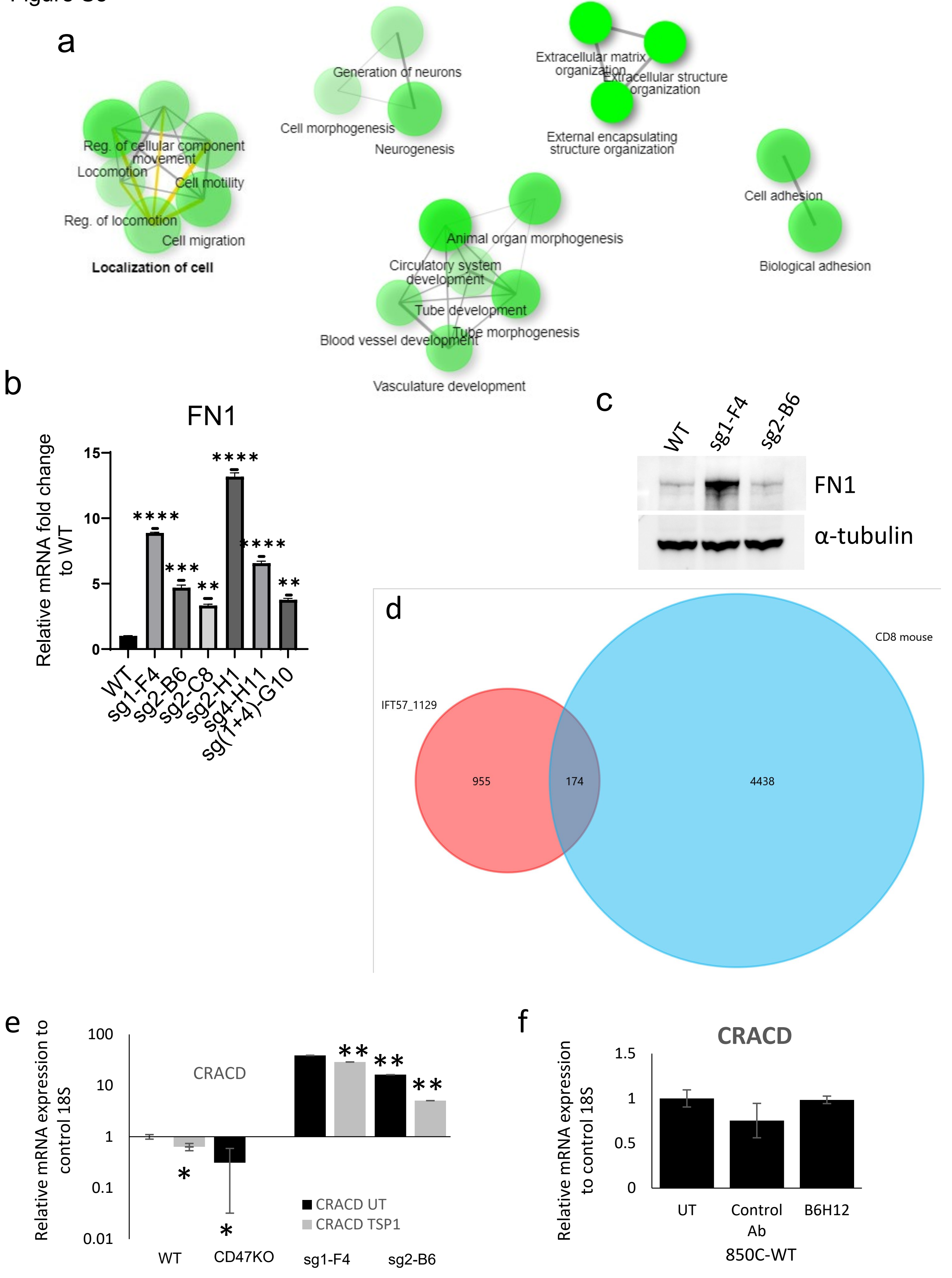
a
Reg. of cellular component movement
Locomotion
Cell motility
Reg. of locomotion
Cell migration
Localization of cell
Generation of neurons
Cell morphogenesis
Neurogenesis
Extracellular matrix organization
Extracellular structure organization
External encapsulating structure organization
Animal organ morphogenesis
Circulatory system development
Tube development
Tube morphogenesis
Blood vessel development
Vasculature development
Cell adhesion
Biological adhesion
b
FN1
Relative mRNA fold change to WT
WT
sg1-F4
sg2-B6
sg2-C8
sg2-H1
sg4-H11
sg(1+4)-G10
c
WT
sg1-F4
sg2-B6
FN1
α-tubulin
d
CD8 mouse
IFT57_1129
955
174
4438
e
Relative mRNA expression to control 18S
CRACD
CRACD UT
CRACD TSP1
WT
CD47KO
sg1-F4
sg2-B6
f
CRACD
Relative mRNA expression to control 18S
UT
Control Ab
B6H12
850C-WT

Figure S6

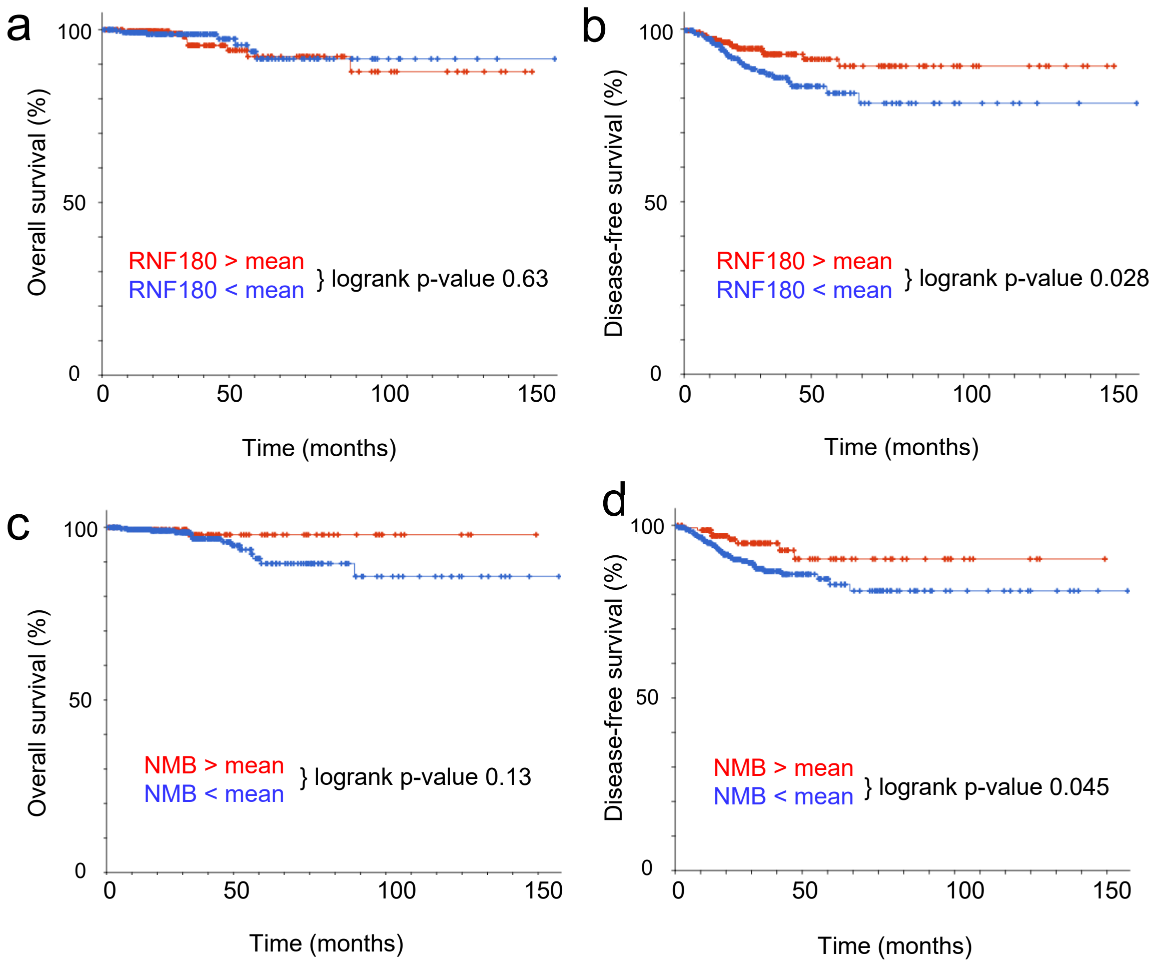
a
b
c
d
Overall survival (%)
Disease-free survival (%)
Time (months)
0
50
100
150
RNF180 > mean
RNF180 < mean
} logrank p-value 0.63
} logrank p-value 0.028
NMB > mean
NMB < mean
} logrank p-value 0.13
} logrank p-value 0.045

Figure S7

a Human IFT57 sgRNA targeting sequence

sgRNA-1 5’-CGTCGTCACGACGTCGGGTT-3’

sgRNA-2 5’-CAAACCCGACGTCGTGACGA-3’

sgRNA-4 5’- TACAGCCACGACCGGTTACC-3’

Human CD47 sgRNA targeting sequence

sgRNA-Intron 5’-CGACCGCCGCCGCGTCACAGG-3’

sgRNA-Extron 5’CAGCAACAGCGCCGCTACCAGGG-3’

b PCR Primers

Sequence Name	Sequence
IFT57 forward	CAC TTC TGT GCT GGT GCA TT
IFT57 reverse	GTG GAA CGT GTA CTA CCG CA
CD47 forward	AGA ATG CTA CTG GCC TTG GT
CD47 reverse	GGA GGT TAA TCC AAT CGC TGT
FN1 forward	ATG CTT TGG AGG CCA GCG GGG CT
FN1 reverse	TGG CCA GTA GTG CCT TCG GGA C
CD47 short forward	GGT TTG AGT ATC TTA GCT CTA GCA C
CD47 short reverse	CCA TCA CTT CAC TTC AGT CAG TTA TTC C
CD47 long forward	GGT TTG AGT ATC TTA GCT CTA GCA C
CD47 long reverse	TCT ACA GCT TTC CTA GGA GG
Forward Primer_KIAA1211	GAG GAT CTG TTC CTG ACC AGT
Reverse Primer_KIAA1211	GGA CTT AGA GAA CTT GGC GTA TC
Forward Primer_NID2	CCG GTG CTG TCG TCG TTA C
Reverse Primer_NID2	GGC TTC GTA GAA GTG CAG GG
Forward Primer_GAS6	GGT AGC TGA GTT TGA CTT CCG
Reverse Primer_GAS6	GAC AGC ATC CCT GTT GAC CTT
Forward Primer_RNF180	GAA GCC CTT CCA GAA TGG ATA AG
Reverse Primer_RNF180	CCC ACA GAA AGG ACA ATT CAG TT
Forward primer_NMB	CCC CAA ATC CAG GAG GCT G
Reverse primer_NMB	TAA GCC ACG CTG TTG TGT CT
