# Supplementary material for: CD47 and IFT57 Are Colinear Genes That Are Highly Coexpressed in Most Cancers and Exhibit Parallel Cancer-Specific Correlations with Survival
Source: Int J Mol Sci. 2024 Aug 17;25(16):8956. doi: 10.3390/ijms25168956 (PMC11354933; doi:10.3390/ijms25168956)

Primary western blot images

Tubulin for FN1 blot (Figure 4)

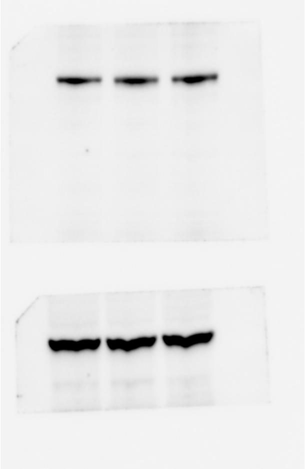

FN1 blot

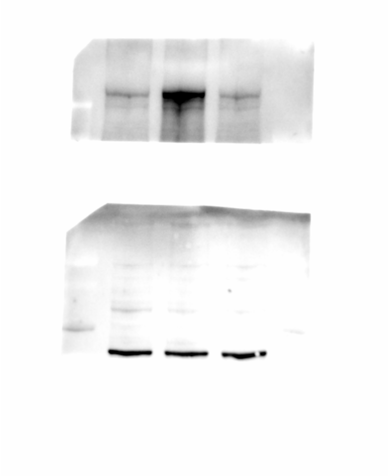

Replicate experiment for FN1 blot

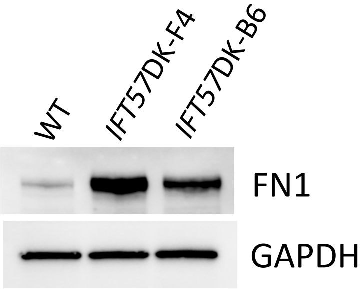

IFT57 blot (Figure 3)

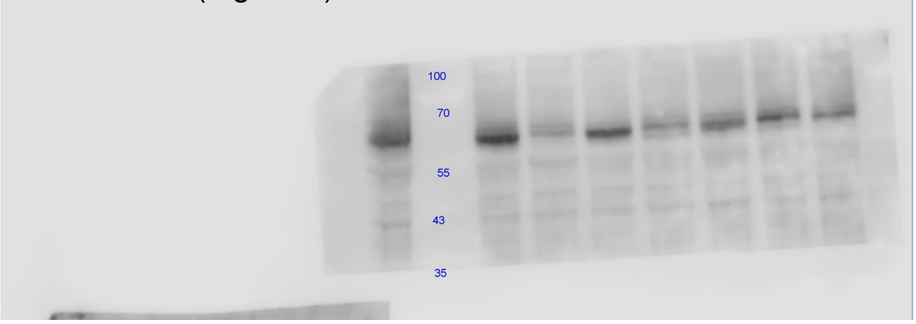

Beta actin for IFT57 blot

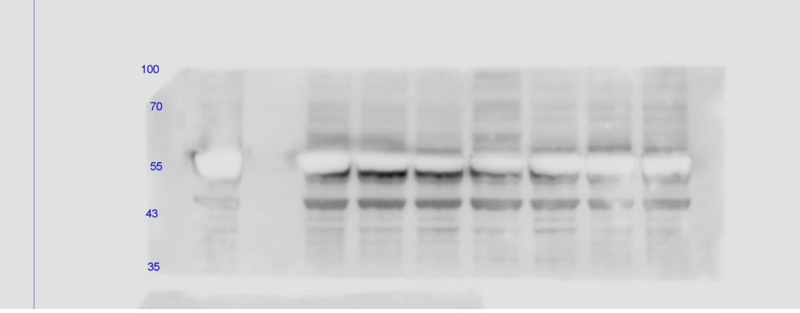

Supplement: Supplementary file 1 [file ijms-25-08956-s001.zip › primary blot images.pdf]
